# Androgen Excess Produces Systemic Oxidative Stress and Predisposes to β-Cell Failure in Female Mice

**DOI:** 10.1371/journal.pone.0011302

**Published:** 2010-06-24

**Authors:** Suhuan Liu, Guadalupe Navarro, Franck Mauvais-Jarvis

**Affiliations:** 1 Division of Endocrinology, Metabolism and Molecular Medicine, Department of Medicine, Feinberg School of Medicine, Northwestern University, Chicago, Illinois, United States of America; 2 Northwestern Comprehensive Center on Obesity, Feinberg School of Medicine, Northwestern University, Chicago, Illinois, United States of America; Louisiana State University, United States of America

## Abstract

In women, excess production of the male hormone, testosterone (T), is accompanied by insulin resistance. However, hyperandrogenemia is also associated with β-cell dysfunction and type 2 diabetes raising the possibility that androgen receptor (AR) activation predisposes to β-cell failure. Here, we tested the hypothesis that excess AR activation produces systemic oxidative stress thereby contributing to β-cell failure. We used normal female mice (CF) and mice with androgen resistance by testicular feminization (Tfm). These mice were exposed to androgen excess and a β-cell stress induced by streptozotocin (STZ). We find that following exposure to T, or the selective AR-agonist dehydrotestosterone (DHT), CF mice challenged with STZ, which are normally protected, are prone to β-cell failure and insulin-deficient diabetes. Conversely, T-induced predisposition to β-cell failure is abolished in Tfm mice. We do not observe any proapoptotic effect of DHT alone or in the presence of H_2_O_2_ in cultured mouse and human islets. However, we observe that exposure of CF mice to T or DHT provokes systemic oxidative stress, which is eliminated in Tfm mice. This work has significance for hyperandrogenic women; excess activation of AR by testosterone may provoke systemic oxidative stress. In the presence of a prior β-cell stress, this may predispose to β-cell failure.

## Introduction

The association between hyperandrogenicity and diabetes in women has been known for almost a century [1], [2]. Indeed, women with polycystic ovarian syndrome (PCOS) develop insulin resistance independently of obesity [3]. Furthermore, testosterone (T) infusion in healthy women decreases insulin-stimulated whole body glucose uptake [4]. The role of excess T in promoting skeletal muscle insulin resistance is also well established from studies in female rodents [5], [6]. Women with hyperandrogenemia, however, show various degrees of pancreatic β-cell dysfunction [7], [8], [9], [10]. In some studies of women with PCOS, β-cell dysfunction was closely associated to the degree of androgenicity, independently of insulin resistance, raising the possibility that excess T may predispose to secondary β-cell failure [9], [10]. Consistent with this hypothesis, in mice, T accelerates the hyperglycemic decompensation in experimental models of insulin-dependent diabetes in which β-cell destruction is induced by oxidative stress or inflammation [11], [12], [13], [14]. Thus, excess T in women may exacerbate the deleterious effect of oxidative stress. Indeed, T provokes oxidative stress in cultured prostate cancer cells [15] and conversely, the AR antagonist flutamide protects against liver injury following trauma-hemorrhage in male by reducing oxidative stress [16]. In addition, T overload in rats increases reactive oxygen species-induced oxidative damage and lipid peroxidation in muscle [17]. Finally, hyperandrogenemia in women with PCOS is accompanied with systemic oxidative stress [18]. Thus, T may provoke systemic oxidative stress which may act as a second hit to provoke β-cell failure in predisposed individuals. T exerts its actions via a ligand activated transcription factor, namely, the androgen receptor (AR). The extent to which the AR plays a role in systemic oxidative stress and β-cell failure in female rodents with hyperandrogenemia is unknown.

Here, we studied the role of androgen and the AR on β-cell survival and systemic oxidative stress in littermate control females (CF) and androgen receptor deficient mice with testicular feminization (Tfm). These mice were exposed to excess T with or without oxidative stress induced by streptozotocin (STZ).

## Results

### AR activation predisposes to STZ-induced diabetes in female mice

**Figure 1:**
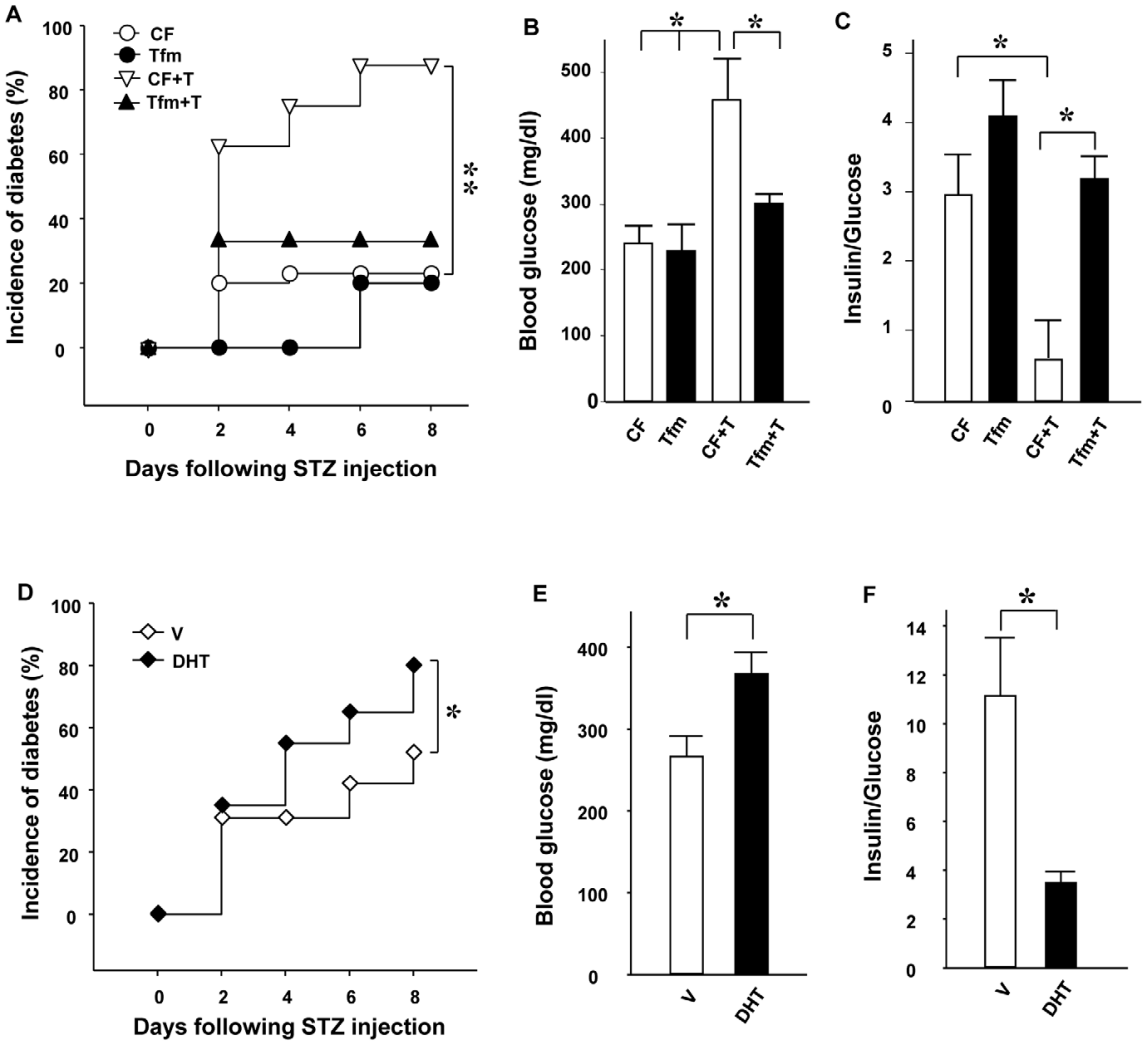
Excess AR activation exacerbates STZ-induced diabetes in mice. (**A**) Cumulative incidence of diabetes (random-fed blood glucose >250 mg/dl) in CF and Tfm treated with V or T (n = 10−15) after STZ challenges (150 mg/kg). (**B**) Random-fed blood glucose in V or T treated mice was measured after STZ injection (day 8). (**C**) The ratio of random-fed insulin (pg/ml)/glucose (mg/dl) in vehicle or T treated mice at day 8 was used as an index of insulin deficiency. (**D**) Cumulative incidence of diabetes in CF and CF treated with DHT mice (n = 10−15) after STZ challenges (150 mg/kg). (**E**) Random-fed blood glucose was measured after STZ injection (day 8) in V- or DHT-treated mice. (**F**) The ratio of random-fed insulin (pg/ml)/glucose (mg/dl) at day 8 in V- or DHT-treated mice. Values represent the mean ± SE. ^*^ P<0.05. V = vehicle.

**Figure 2:**
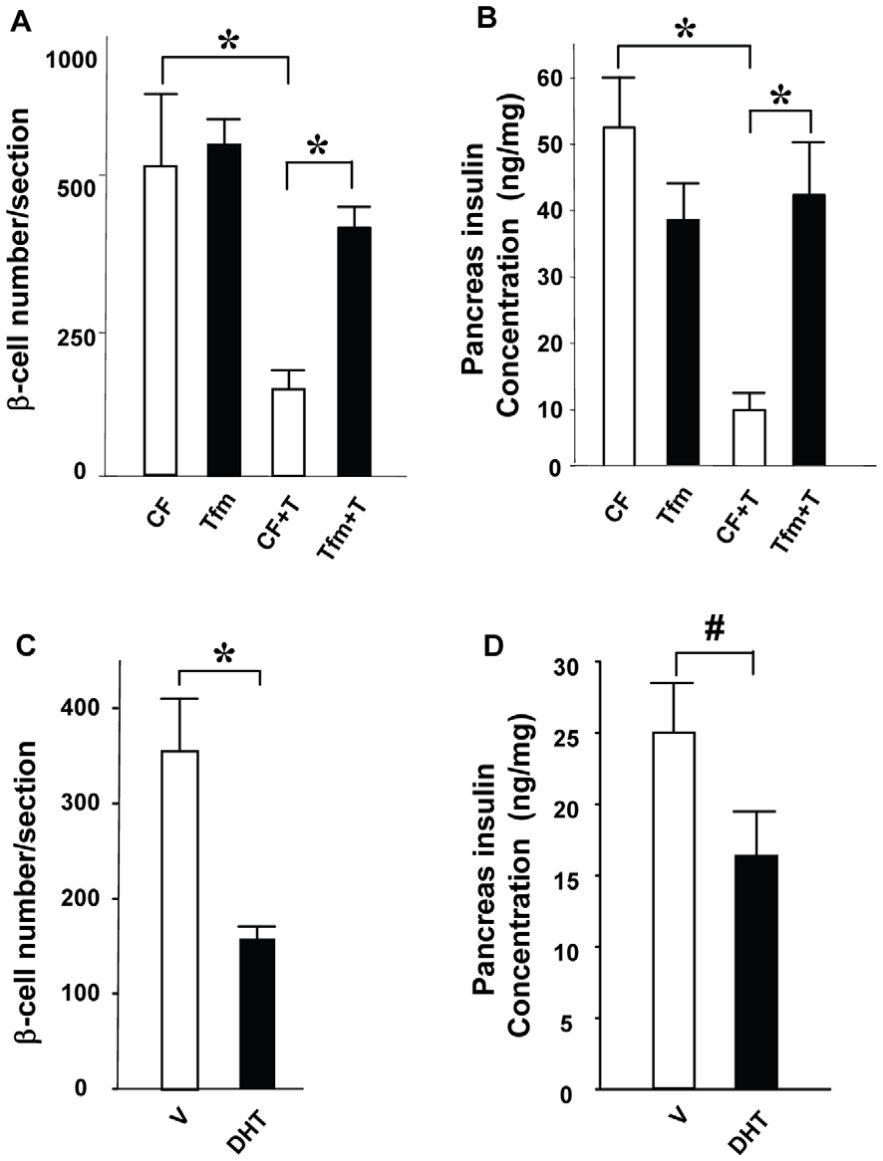
Excess AR activation exacerbated STZ-induced β-cell loss in mice. (**A**) β-cell number per section from CF and Tfm treated with V or T. (**B**) Pancreatic insulin concentration in mice from (A). (**C**) β-cell number in CF treated with V or DHT-treated mice. (D) Pancreas insulin content in mice from (C). Values represent the mean ± SE. ^*^ P<0.05, #P = 0.08. V = vehicle.

To produce a β-cell stress *in vivo*, we used a single high-dose injection of STZ (150 mg/kg). Females are partially protected from STZ induced- stress and β-cell failure because of estradiol protection [19], [20]. In order to address the effects of AR activation in β-cell death, we compared littermate normal control and AR-deficient mice with the testicular feminizing (Tfm) mutation. The Tfm mouse is a well-established mouse model of total androgen resistance with a mutant and inactive AR [21], [22], [23]. Because AR gene is on the X chromosome, females are heterozygous carrier and genetic male are phenotypically females and comparable to females. We treated the mice with vehicle (V), T, or the non-aromatizable and pure AR agonist, dihydrotestosterone (DHT). Following STZ challenge, control female (CF) mice exposed to V were protected by endogenous estradiol and showed minimal predisposition from STZ-induced insulin-deficient diabetes (Fig. 1 A–C). Conversely, CF mice exposed to T (Fig. 1A–C) or DHT (Fig. 1D–F,) became vulnerable to STZ and showed a severe predisposition to insulin-deficient diabetes. We observed that, both in normal condition and following T exposure, Tfm mice were protected against STZ-induced insulin deficient diabetes (Fig. 1A–C). Consistent with the results shown previously, CF mice exposed to STZ only exhibited minimal decrease in β-cell number and pancreas insulin concentrations when exposed to T (Fig. 2A, B). Conversely, CF mice exposed to T or DHT exhibited a dramatic loss of β-cell number and pancreas insulin concentration (Fig. 2A–D). We found that Tfm mice were protected from the deleterious effect of T on β-cell destruction; they retained a normal β-cell number and pancreas insulin concentration following exposure to STZ (Fig. 2 A, B).

### AR activation does not provoke inflammation or apoptosis in female islets

**Figure 3:**
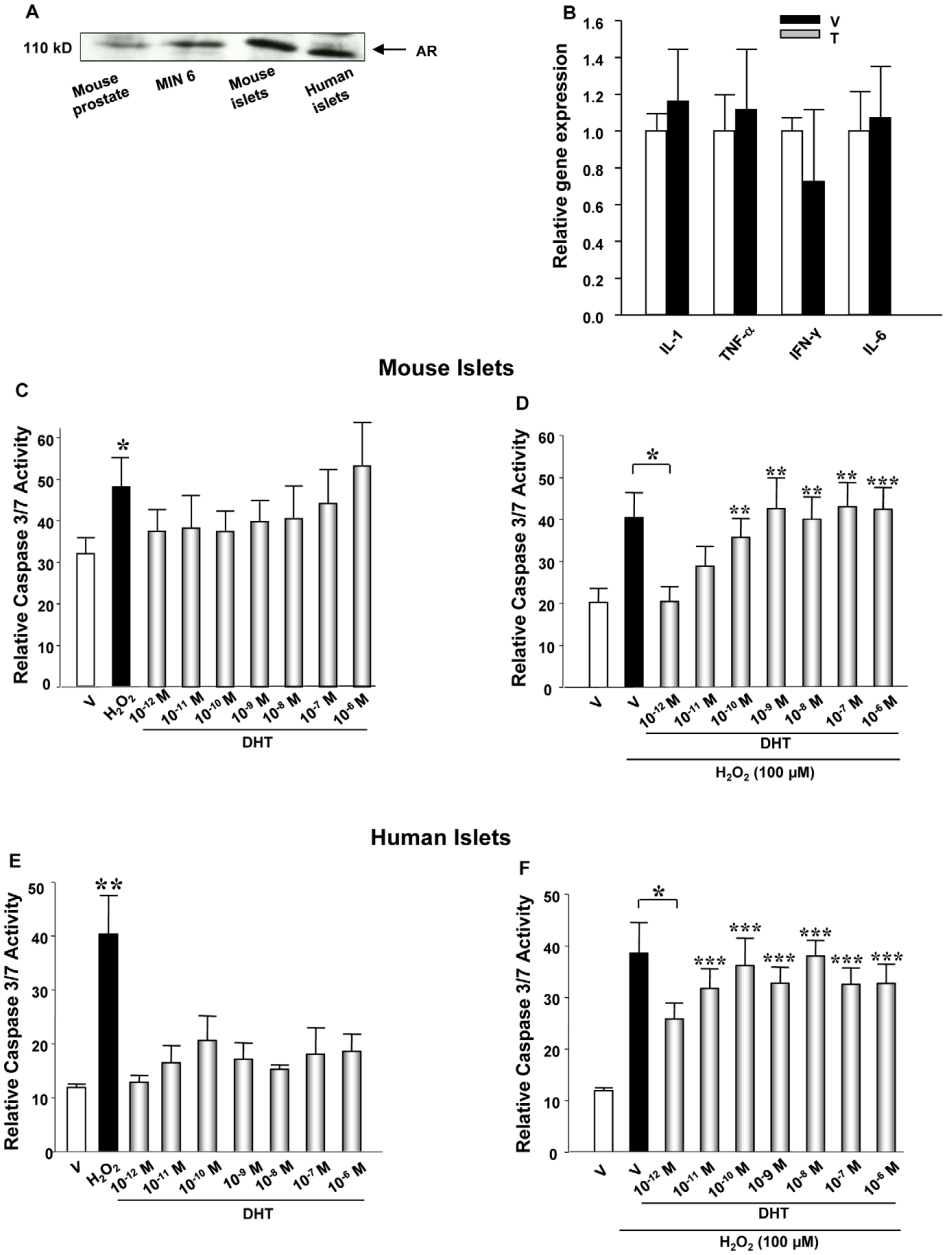
Excess AR activation in islets does not provoke inflammation or apoptosis. (**A**) AR expression in pancreatic islets was measured by western blotting. (**B**) Relative gene expression of proinflammatory cytokines (PIC) in the islets. (**C**) Caspase 3/7 activity was measured in female mouse islets by luminescence following DHT dose-response treatment at the indicated concentrations. (**D**) Caspase 3/7 activity was measured in female mouse islets by luminescence following exposure to H_2_O_2_ (100 µM) and DHT dose-response treatment at the indicated concentrations. (**E**) Caspase 3/7 activity was measured in human islets as indicated in (C). (**F**) Caspase 3/7 activity was measured in human islets as indicated in (D). Values represent the mean ±SE. ^*^ P<0.05, **P<0.01, ***P<0.001 each condition vs. vehicle only treatment.

Pro-inflammatory cytokines (PIC), and in particular IL-1β, are thought to be important pathogenic effectors responsible for the induction of β-cell dysfunction and apoptosis in both type 1 and type 2 diabetes [24]. Massive induction of apoptosis in β-cells can be elicited in vitro by a combination of IL-1β plus IFNγ and/or TNFα [24], [25]. Because AR is a ligand-activated transcription factor expressed in islets (Fig. 3A), we looked at the possibility that T increases PIC gene expression from islets. However, we observed no effect of T on PIC gene expression in islets from wild type (WT) mice treated by T (Fig. 3B), eliminating the existence of a local T-induced inflammation leading to β-cell destruction. Since T does not provoke inflammation in the islet *in vivo*, we tested the possibility that direct islet exposure to excess androgen provokes apoptosis. The pro-apoptotic effect of DHT was thus investigated in cultured female mouse and human islets using a luminescent assay for activated caspase-3, the “executioner” of apoptosis. In a dose-response experiment, we did not observe that DHT, even used at pharmacological concentrations, increased caspase 3 activity, eliminating a direct pro-apoptotic effect of DHT alone on β-cell survival *in vitro* (Fig. 3C and E). Finally, we looked at the possibility that, as observed *in vivo* following STZ challenge, DHT exacerbates the pro-apoptotic effect of oxidative stress induced by H_2_O_2_. H_2_O_2_ provoked a significant increase in caspase 3 activity in mouse and human islets, and interestingly this was prevented by DHT at low physiological concentration (10^−12^M) (Fig. 3D and F). Conversely, the pro-apoptotic effect of H_2_O_2_ was not increased by DHT at concentrations ranging from physiological to pharmacological (Fig. 3D and F). Thus, excess AR activation in cultured islets does not increase the susceptibility to H_2_O_2_-induced apoptosis.

### AR activation provokes systemic oxidative stress in female mice

**Figure 4:**
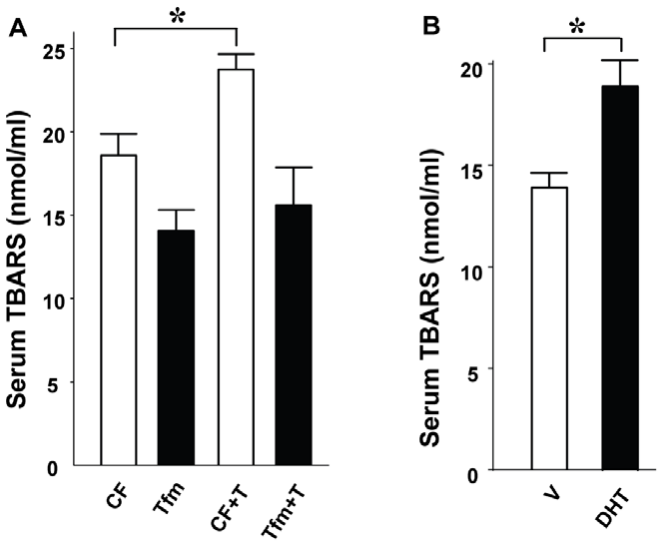
Excess AR activation provokes systemic oxidative stress. (A) Plasma TBARS concentrations were measured in CF and Tfm mice treated with V or T. (B) TBARS level in CF treated with V or DHT mice. Values represent the mean ± SE. ^*^ P<0.05.

**Figure 5:**
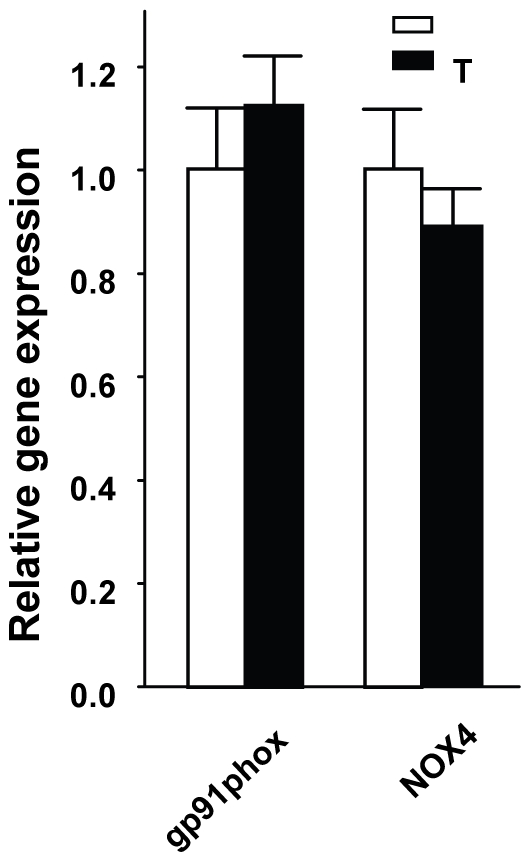
Testosterone does not affect the expression of NOX subunits in WAT. Gene expression of gp91^phox^ and NOX4 in perigonadal fat pads from CF treated with V or T. Values represent the mean ± SE. ^*^ P<0.05.

Since T exacerbates STZ-induced islet destruction but does not provoke β-cell apoptosis by itself, we reasoned that T may increase systemic oxidative stress. Indeed, women with hyperandrogenism show systemic oxidative stress [18]. Thus, we looked at the effect of AR activation on systemic markers of oxidative stress in CF and Tfm mice. Oxidative stress in organisms results in the peroxidation of all major biomolecules, such as DNA, proteins, and lipids. To measure oxidative stress, we quantified lipid peroxidation in serum using the classical thiobarbituric acid reactive substances (TBARS) method [26]. In basal conditions, we observed a minor decrease in serum TBARS concentrations in Tfm compared to CF mice, T and DHT exposure produced an increased in serum TBARS concentrations in CF mice (Fig. 4A and B), but this effect was not observed in Tfm mice (Fig. 4A). Systemic oxidative stress may originate from the NADPH oxidase (NOX) complex from accumulated fat [27]. NOX is a major source of reactive oxygen species (ROS) in various cells. We quantified the expression of gp91^phox^ and Nox4, a homolog of gp91^phox^, which is specific to white adipose tissue (WAT) [28] and is not expressed in macrophages [29]. None of these genes were elevated (Fig. 5) ruling out the possibility that oxidative stress comes from fat.

## Discussion

We show that excess T provokes systemic oxidative stress and, in synergism with a concomitant β-cell injury, predisposes to β-cell failure in female mice via AR-dependent mechanisms.

Paradoxically, in the non-obese diabetic (NOD) type 1 diabetic model, the female predisposition to diabetes is prevented by androgens [30]. The NOD mouse, however, is the only model of β-cell failure that does not exhibit the classical male predominance that characterizes rodents and humans [31]. The gender dimorphism in NOD mice is related to an enhanced target organ-specific estrogen and androgen sensitivity of lymphocyte in [32], [33] and a specific gene that segregate with diabetes and modulates a target organ-specific androgen sensitivity [34]. Therefore, the NOD model, although excellent for studying autoimmune β-cell death is not appropriate for studying the role of sex hormones in islet biology and diabetes.

To determine whether excess AR activation exacerbates the susceptibility to a β-cell stress *in vivo*, we used the STZ mouse model. STZ does not provoke β-cell dysfunction as encountered in women with PCOS. However, STZ provokes an increase in islet ROS leading to cellular damage and apoptosis and is a useful pharmacological tool to mimic an *in vivo* islet stress induced by hyperglycemia or cytokines in diabetes [35], [36]. We have successfully used the STZ model of gender dimorphism to show that estrogen favor islet survival through at least three estrogen receptors [19], [20]. T overload increases oxidative stress, ROS-induced oxidative damage, and lipid peroxidation in vitro and in vivo [15], [16], [17]. In addition, women with hyperandrogenism have systemic oxidative stress [18] and β-cell dysfunction [7], [8], [9], [10]. Here, we show for the first time that T and DHT produce systemic oxidative stress in mice through an AR-dependent mechanism. This is associated to an increased severity of β-cell destruction in female mouse islets challenged with STZ. Since STZ produces an islet stress, AR-induced systemic oxidative stress may synergize with STZ to act as a second hit, aggravating β-cell injury. However, excess AR activation without prior β-cell injury is not sufficient since T infusion in healthy women does not produce β-cell dysfunction [4] .Thus, in women with a prior β-cell defect, excess T may predispose to β-cell failure through the cumulative action of various β-cell stresses including insulin resistance and circulating oxidative stress.

The question that remains unanswered is whether direct AR activation in β-cells alters survival and function *in vivo*. In dopaminergic neurons [37] and endothelial cells, T provokes apoptosis. T induction of the AR in endothelial cells increases in NF-κB nuclear translocation and pro-inflammatory gene expression [38] and T enhances apoptotic damage in human endothelial cells cultured in stress conditions [39]. In mice, however, T excess does not increase islet pro-inflammatory cytokines production ruling out an effect of AR in producing local islet inflammation. In addition, in cultured mouse and human islets, DHT does not show a significant pro-apoptotic effect and does not exacerbate the susceptibility to another β-cell stressor like oxidative stress, event at pharmacological concentrations. This suggests that if excess AR action in β-cells impairs survival *in vivo*, AR must also be activated in non-islets tissues to promote β-cell stress via a circulating factor or neuronal input on islets. Studies are ongoing to determine the role of AR in the predisposition to β-cell failure in females *in vivo*.

What is the origin of systemic oxidative stress in hyperandrogenic mice? Systemic oxidative stress may originate from accumulated fat [27], or androgen-stimulated circulating mononuclear cells (MNC). Against the involvement of WAT, is the observation that WAT mass and the expression of molecular markers of NOX, a major source of ROS in various cells, are not increased in our hyperandrogenic mice. With regard to the second possibility, ROS generation from MNCs is increased in PCOS women independent of obesity and correlated to testosterone levels [18]. Thus, excess androgen in females may provoke systemic oxidative stress via AR action on MNC. Further studies are needed to determine the role of AR in β-cells and MNCs in the predisposition to β-cell failure in female mice.

## Materials and Methods

### Animals

Mice carrying the Tfm mutation on a C57BL/6 background and C57BL/6 mice were purchased from Jackson Laboratory. Testicular feminization (*AR^Tfm^*) is a dominant spontaneous mutation on the X chromosome. Females carry the tabby mutation (Ta) on the other X chromosome (Tfm/Ta). Since the tabby gene is closely linked to the Tfm mutation, mating between Ta/Tfm females and Ta/Y males were used to generate carrier female (Tfm/Ta), affected male (Tfm/Y), and normal male and female (Ta/Y and Ta/Ta) offspring. All protocols for animal use and euthanasia were approved by the Animal Care Use Committee of Northwestern University, in accordance with NIH guidelines.

### Androgen infusion and induction of experimental diabetes

T, DHT and vehicle (cholesterol) were administered by subcutaneous 21-day release pellets (5 mg/pellet, Innovative research of America). One week after pellet insertion, a group of 8-week-old mice were exposed to a single intraperitoneal (IP) injection of 150 mg/kg of STZ to induce diabetes as described [19]. Blood glucose was measured every 48 h following STZ injection. At day 8 following STZ injection, serum was collected for measurement of insulin concentrations, mice were sacrificed, and pancreases were processed for measurement of pancreas insulin concentration.

### Pancreas insulin concentration

Whole pancreas was weighed and homogenized in acid/ethanol. Pancreas homogenates were centrifuged and their supernatant was used to measure pancreas insulin concentration as previously described [19].

### Islet isolation

Female mouse islets were isolated by collagenase digestion as described [19]. Briefly, the pancreas was injected through the pancreatic duct with 2 ml of 2 mg/ml collagenase (Sigma) in Hanks' buffered saline solution (HBSS), dissected out, incubated at 37°C for 15 min, and passed through a 400-mm wire mesh. The digested pancreas was rinsed with HBSS, and islets were separated by density gradient in Histopaque (Sigma). After several washes with HBSS and PBS, islets were then either cultured in phenol-red free RPMI medium containing 11 mM glucose, 10% charcoal stripped FBS, 1 mM glutamine, penicillin (100 U/ml), streptomycin (100 µg/ml) for DHT treatment study or frozen in liquid nitrogen and then stored at −80°C for RNA extraction.

### DHT stimulation and caspase 3/7 activity assay

Islets were cultured in phenol-red free RPMI medium containing 11 mM glucose, 10% charcoal stripped FBS. Islets were treated with DHT at the dose-response of 10^−12^M, 10^−11^M, 10^−10^M, 10^−9^M, 10^−8^M, 10^−7^M, or 10^−6^M for 48 h in the presence or absence of H_2_O_2_ (100 µM) for 5 hours. Ethanol was used as the vehicle. Medium was changed daily and DHT was added daily. Following 48 h DHT treatment, Caspase activity was measured using Caspase-Glo 3/7 assay kit (Promega) as described [19], [20]. Briefly, islets were collected and centrifuged for 5 min at 1200 rpm, washed with PBS and transferred to a 96-well plate in a 100 µl volume. Islets were then lysed with 100 µl Caspase-Glo 3/7 reagent and incubated at room temperature for 60 min. Luciferase activity was measured using a *Synergy*™ *2* Multi-Mode Microplate Reader *(*BioTek). Values are reported as relative luciferase units corrected for total protein concentration.

### Real time RT-PCR

Total RNA was prepared from islets Trizol (Invitrolgen) following the manufacturer's instructions. 1((g of total RNA was reverse transcripted to 20((l cDNA using iScript cDNA Synthesis Kit (BIO-RAD. Real time PCR was performed using iQTMSYBR®Green Supermix (BIO-RAD). The mRNA expression was studied by using specific oligonucleotides primers. The threshold cycle values (C_T_) were determined as a measure of the cycle number at which a statistically significant increase in fluorescence intensity is first detected. Beta actin was used as an endogenous control to account for possible variations due to differences in initial RNA quality and the efficiency of the cDNA synthesis reaction. The abundance of the amplified DNA was then determined from the C_T_ values and was normalized to the value for the control gene beta actin to yield the relative abundance. When comparing samples from two or more experimental groups, the relative abundance values were normalized to the average relative abundance for the control female group and the resulting ratios were presented in figures. The oligonucleotide primers used were as follows: IL-1β (forward): 5′-AGCTCTCCACCTCAATGGACAGAA-3′, (Reverse) -5′ ATTGCTTGGGATCCACACTCTCCA-3′; TNF-α (forward): 5′-GTC CCC AAA GGG ATG AGA AGT-3′, (reverse) 5′-TGA GAT AGC AAA TCG GCT GAC-3′; IFN-γ: (forward) 5′-ACT GGC AAA AGG ATG GTG AC-3′, (reverse) 5′-TGA GCT CAT TGA ATG CTT GG-3′; IL-6: (forward) 5′-ACAACCACGGCCTTCCCTACTT-3′, (reverse) 5′-CACGATTTCCCAGAGAACATGTG-3′; gp91^phox^ (forward): 5′-TTGGGTCAGCACTGGCTCTG-3′, (reverse) 5′-TGGCGGTGTGCAGTGCTATC-3′; NOX4: (forward) 5′- TGTTGGGCCTAGGATTGTGTT-3′, (reverse) 5′- AAAAGGATGAGGCTGCAGTTG-3′.

### TBARS assay

Systemic oxidative stress was assessed by measuring the concentration of thiobarbituric acid reactive substances (TBARS) in plasma [26] according to the manufacturer's instructions (Zeptometrix).

### Statistical analysis

Results are presented as mean ± SE as specified in figures. All statistical analyses were performed using the unpaired Student's *t* test. Cumulative incidence of diabetes was determined by Kaplan-Meier estimates and statistical analysis of differences was determined by log-rank test. A value of p<0.05 was considered significant.

## References

[1] Achard C, Thiers J (1921). Le virilisme pilaire et son association a l'insuffisance glycolytique (diabete des femmes a barbe).. Bull Acad Natl Med Paris.

[2] Stein J, Leventhal ML (1935). Amenorreha associated with bilateral polycystic ovaries.. Am J obstet Gynecol.

[3] Dunaif A, Segal KR, Futterweit W, Dobrjansky A (1989). Profound peripheral insulin resistance, independent of obesity, in polycystic ovary syndrome.. Diabetes.

[4] Diamond MP, Grainger D, Diamond MC, Sherwin RS, Defronzo RA (1998). Effects of methyltestosterone on insulin secretion and sensitivity in women.. J Clin Endocrinol Metab.

[5] Holmang A, Svedberg J, Jennische E, Bjorntorp P (1990). Effects of testosterone on muscle insulin sensitivity and morphology in female rats.. Am J Physiol.

[6] Holmang A, Larsson BM, Brzezinska Z, Bjorntorp P (1992). Effects of short-term testosterone exposure on insulin sensitivity of muscles in female rats.. Am J Physiol.

[7] O'Meara NM, Blackman JD, Ehrmann DA, Barnes RB, Jaspan JB (1993). Defects in beta-cell function in functional ovarian hyperandrogenism.. J Clin Endocrinol Metab.

[8] Dunaif A, Finegood DT (1996). Beta-cell dysfunction independent of obesity and glucose intolerance in the polycystic ovary syndrome.. J Clin Endocrinol Metab.

[9] Holte J, Bergh T, Berne C, Berglund L, Lithell H (1994). Enhanced early insulin response to glucose in relation to insulin resistance in women with polycystic ovary syndrome and normal glucose tolerance.. J Clin Endocrinol Metab.

[10] Goodarzi MO, Erickson S, Port SC, Jennrich RI, Korenman SG (2005). beta-Cell function: a key pathological determinant in polycystic ovary syndrome.. J Clin Endocrinol Metab.

[11] Rossini AA, Williams RM, Appel MC, Like AA (1978). Sex differences in the multiple-dose streptozotocin model of diabetes.. Endocrinology.

[12] Morrow PL, Freedman A, Craighead JE (1980). Testosterone effect on experimental diabetes mellitus in encephalomyocarditis (EMC) virus infected mice.. Diabetologia.

[13] Maclaren NK, Neufeld M, McLaughlin JV, Taylor G (1980). Androgen sensitization of streptozotocin-induced diabetes in mice.. Diabetes.

[14] Paik SG, Michelis MA, Kim YT, Shin S (1982). Induction of insulin-dependent diabetes by streptozotocin. Inhibition by estrogens and potentiation by androgens.. Diabetes.

[15] Ripple MO, Hagopian K, Oberley TD, Schatten H, Weindruch R (1999). Androgen-induced oxidative stress in human LNCaP prostate cancer cells is associated with multiple mitochondrial modifications.. Antioxid Redox Signal.

[16] Kan WH, Hsieh CH, Schwacha MG, Choudhry MA, Raju R (2008). Flutamide protects against trauma-hemorrhage-induced liver injury via attenuation of the inflammatory response, oxidative stress, and apopotosis.. J Appl Physiol.

[17] Pansarasa O, D'Antona G, Gualea MR, Marzani B, Pellegrino MA (2002). “Oxidative stress”: effects of mild endurance training and testosterone treatment on rat gastrocnemius muscle.. Eur J Appl Physiol.

[18] Gonzalez F, Rote NS, Minium J, Kirwan JP (2006). Reactive oxygen species-induced oxidative stress in the development of insulin resistance and hyperandrogenism in polycystic ovary syndrome.. J Clin Endocrinol Metab.

[19] Le May C, Chu K, Hu M, Ortega CS, Simpson ER (2006). Estrogens protect pancreatic beta-cells from apoptosis and prevent insulin-deficient diabetes mellitus in mice.. Proc Natl Acad Sci U S A.

[20] Liu S, Le May C, Wong WP, Ward RD, Clegg DJ (2009). Importance of extranuclear estrogen receptor-alpha and membrane G protein-coupled estrogen receptor in pancreatic islet survival.. Diabetes.

[21] Lyon MF, Hawkes SG (1970). X-linked gene for testicular feminization in the mouse.. Nature.

[22] Charest NJ, Zhou ZX, Lubahn DB, Olsen KL, Wilson EM (1991). A frameshift mutation destabilizes androgen receptor messenger RNA in the Tfm mouse.. Mol Endocrinol.

[23] Murphy L, Jeffcoate IA, O'Shaughnessy PJ (1994). Abnormal Leydig cell development at puberty in the androgen-resistant Tfm mouse.. Endocrinology.

[24] Donath MY, Storling J, Maedler K, Mandrup-Poulsen T (2003). Inflammatory mediators and islet beta-cell failure: a link between type 1 and type 2 diabetes.. J Mol Med.

[25] Mandrup-Poulsen T (2001). beta-cell apoptosis: stimuli and signaling.. Diabetes.

[26] Armstrong D, Browne R (1994). The analysis of free radicals, lipid peroxides, antioxidant enzymes and compounds related to oxidative stress as applied to the clinical chemistry laboratory.. Adv Exp Med Biol.

[27] Furukawa S, Fujita T, Shimabukuro M, Iwaki M, Yamada Y (2004). Increased oxidative stress in obesity and its impact on metabolic syndrome.. J Clin Invest.

[28] Mahadev K, Motoshima H, Wu X, Ruddy JM, Arnold RS (2004). The NAD(P)H oxidase homolog Nox4 modulates insulin-stimulated generation of H2O2 and plays an integral role in insulin signal transduction.. Mol Cell Biol.

[29] Sorescu D, Weiss D, Lassegue B, Clempus RE, Szocs K (2002). Superoxide production and expression of nox family proteins in human atherosclerosis.. Circulation.

[30] Fox HS (1992). Androgen treatment prevents diabetes in nonobese diabetic mice.. J Exp Med.

[31] Yin N, Wang D, Zhang H, Yi X, Sun X (2004). Molecular mechanisms involved in the growth stimulation of breast cancer cells by leptin.. Cancer Res.

[32] Pearce RB, Formby B, Healy K, Peterson CM (1995). Association of an androgen-responsive T cell phenotype with murine diabetes and Idd2.. Autoimmunity.

[33] Bao M, Yang Y, Jun HS, Yoon JW (2002). Molecular mechanisms for gender differences in susceptibility to T cell-mediated autoimmune diabetes in nonobese diabetic mice.. J Immunol.

[34] Rosmalen JG, Pigmans MJ, Kersseboom R, Drexhage HA, Leenen PJ (2001). Sex steroids influence pancreatic islet hypertrophy and subsequent autoimmune infiltration in nonobese diabetic (NOD) and NODscid mice.. Lab Invest.

[35] Friesen NT, Buchau AS, Schott-Ohly P, Lgssiar A, Gleichmann H (2004). Generation of hydrogen peroxide and failure of antioxidative responses in pancreatic islets of male C57BL/6 mice are associated with diabetes induced by multiple low doses of streptozotocin.. Diabetologia.

[36] Gille L, Schott-Ohly P, Friesen N, Schulte im Walde S, Udilova N (2002). Generation of hydroxyl radicals mediated by streptozotocin in pancreatic islets of mice in vitro.. Pharmacol Toxicol.

[37] Cunningham RL, Giuffrida A, Roberts JL (2009). Androgens induce dopaminergic neurotoxicity via caspase-3-dependent activation of protein kinase Cdelta.. Endocrinology.

[38] Death AK, McGrath KC, Sader MA, Nakhla S, Jessup W (2004). Dihydrotestosterone promotes vascular cell adhesion molecule-1 expression in male human endothelial cells via a nuclear factor-kappaB-dependent pathway.. Endocrinology.

[39] Ling S, Dai A, Williams MR, Myles K, Dilley RJ (2002). Testosterone (T) enhances apoptosis-related damage in human vascular endothelial cells.. Endocrinology.

